# Molecular epidemiology of hcv among health care workers of khyber pakhtunkhwa

**DOI:** 10.1186/1743-422X-8-105

**Published:** 2011-03-08

**Authors:** Sanaullah Khan, Sobia Attaullah, Sultan Ayaz, Shahid Niaz Khan, Sumaira Shams, Ijaz Ali, Muhammad Bilal, Sami Siraj

**Affiliations:** 1 Molecular Parasitology and Virology Laboratory, Department of Zoology, Kohat University Kohat Khyber Pakhtunkhwa, Pakistan; 2 Department of Zoology, Islamia College Peshawar (Chartered University) Peshawar, Pakistan; 3 Institution of Biotechnology and Genetic Engineering, KP Agricultural University Peshawar, Pakistan; 4 Centre for Applied Molecular Biology, Ministry of Science and Technology Lahore, Pakistan; 5 Institute of Basic Medical Sciences, Khyber Medical University Peshawar, Pakistan

## Abstract

**Background:**

Studies of the molecular epidemiology and risk factors for hepatitis C virus (HCV) in health care workers (HCWs) of Peshawar, Khyber Pakhtunkhwa region are scarce. Lack of awareness about the transmission of HCV and regular blood screening is contributing a great deal towards the spread of hepatitis C. This study is an attempt to investigate the prevalence of HCV and its possible association with both occupational and non-occupational risk factors among the HCWs of Peshawar.

**Results:**

Blood samples of 824 HCWs, aged between 20-59 years were analysed for anti-HCV antibodies, HCV RNA and HCV genotypes by Immunochromatographic tests and PCR. All relevant information was obtained from the HCWs with the help of a questionnaire. The study revealed that 4.13% of the HCWs were positive for HCV antibodies, while HCV RNA was detected in 2.79% of the individuals. The most predominant HCV genotype was 3a and 2a.

**Conclusion:**

A program for education about occupational risk factors and regular blood screening must be implemented in all healthcare setups of Khyber Pakhtunkhwa province in order to help reduce the burden of HCV infection.

## Background

Acquisition of blood borne pathogens is a potential occupational health hazard for health care workers (HCWs) across the world [1]. Transmissions of over 20 different pathogens have been reported in HCWs because of occupational exposure [2,3]. Despite of continuous progressive preventive measures and employment of modern medical apparatus, HCWs performing exposures prone procedures run a risk for a vast array of blood born pathogen like Hepatitis C Virus (HCV) [4,5]. HCV, compared to a "viral time bomb, is leading hepatotropic virus and predominant cause of severe pathological consequences like acute hepatitis, chronic liver diseases and hepatocellular carcinoma [6,7].

Currently, approximately 10 million people in Pakistan are suffering from HCV, covering 6% of the overall population and it falls in the intermediate endemic zone [8]. Such a high prevalence of HCV has earlier been attributed to the absence of good preventive measures, inadequate funding for health care resources, tremendous increase in work load, inadequate provision of barrier devices for the HCWs. Moreover, high frequency of HCV among the general population put them at a high risk of acquiring HCV infection [5,9]. Care of HCWs is utmost important because healthy and fearless health worker can provide better services to suffering humanity [10]. A small number of studies are available on the epidemiology of HCV in HCWs from different regions of Pakistan. However in Khyber Pakhtunkhwa, the molecular epidemiology of HCV and its associated occupational risk factors have never been investigated before. This study was designed to know the prevalence of anti-HCV, HCV RNA, HCV genotypes and occupational risk factors for HCV infection among the HCWs in major hospitals of Peshawar, Khyber Pakhtunkhwa Pakistan.

## Methods

### Setting

HCWs from three, major public sector, tertiary and high complexity care hospitals of Peshawar, including Khyber Teaching Hospital, Lady Reading Hospital and Hayatabad Medical Complex Hospital were sources of blood samples for this study.

### Study Population

This study was conducted with the approval of the Medical Superintendents of source hospitals and the ethics committee of Kohat University of Science and Technology (KUST) Kohat Pakistan. The HCWs working for the last six months at the source hospitals were divided into five groups. i- doctors (surgeons, physicians and dentists); ii- nursing staff (staff nurses, professional nurses and midwifery staff); iii- specialized technicians (X-rays, anesthesia and laboratory personals; serologists, microbiologists, hematologists, pathologists, biochemists, biomedical assistants, physiotherapists and occupational therapists), iv- Assistants (radiology, operational services, helpers, attendants) and general services (laboratory, cleaning, laundry, blood bank, pharmacists, security guard housekeeping staff, dispensers, security guards, catering staff and drivers of ambulance) and v- administrative staff.

### Study procedure

Self assessment questionnaire was pre-designed according to the object of the study which included, i) Personal data; gender, marital status, age, and education, ii) Professional data; job category, working department and duration of the job, iii) History of the risk of viral hepatitis infection including blood transfusion, intravenous drug usage, hospitalization, surgery and dental treatment, vi) History of HCV including history of jaundice, history of HCV in the family and v) History of frequency of occupational exposure.

#### Laboratory test

Serum was separated after blood collection, labeled and stored frozen at -20°C at the laboratory of Molecular Parasitology and Virology, Department of Zoology, KUST Kohat. All the samples were screened by immunochromatography tests (Acurate Diagnostics, USA). HCV RNA was isolated with GF-1 RNA extraction Kit (Vivantus, USA), reverse transcribed with 200U of Moloney muriene leukemia virus reverse transcriptase (Fermentas Inc. USA) and 5'UTR of HCV was amplified with nested primers. Positive samples for HCV RNA were genotyped as mentioned elsewhere [11].

### Statistical analysis

Results were statistically analyzed by using Chi square (with Yates's test) or Fisher's exact test. The disease "exposure" association was determined by estimating the Odd ratio (OR) and 95% Confidence intervals (CI) in the case of significant association found. In this study *P *value of less than 0.05 was adopted as statistically significant.

## Results

Among the 824 participated HCWs, 200 (24.2%) were doctors, 240 (29.1%) were nursing staff, 240 (29.1%) were technicians, 114 (13.8%) were general and assistant staff and 30 (3.6%) were administrative staff. The duration of their employment ranged from 6 months to 29 years (mean 14.4 ± 3.21 years) (Table 1). Among them, 493 (59.8%) were male and 331 (40.2%) were female, 677 (82.2%) were married and 147 (17.8%) were unmarried. Overall mean age was 33.8 ± 8.2 years and ranged from 20 to 59 years. 69.4% (572) and 30.6% (252) HCWs fell in undergraduate and graduate categories, respectively. History of blood transfusion was recorded in 24 HCWs (2.91%), dental treatment in 100 HCWs (12.2%), intravenous drug injections in 1 (0.12%), hospitalization in 98 (11.9%), surgery in 31 (3.8%) while none had a positive history in their families. History of HCV positivity was recorded in 6 (0.72%) HCWs.

**Table 1:** HCWs distribution (%) according to age, gender and occupational categories.

Gender	Age (in years)				Total
		
	20-29	30-39	40-49	50-59	
Female	89	169	62	11	**331**

Male	106	171	178	38	**493**

**Categories of HCWs**					

Doctor	68	96	32	4	**200**

Nurse	51	113	73	3	**240**

Technician	48	83	98	11	**240**

Assistant and general staff	27	41	30	16	**114**

Administration staff	1	7	4	18	**30**

**Total**	**195**	**340**	**237**	**52**	**824**

4.1% (34 out of 824) of HCWs had antibodies against HCV, of which 21 (61.8%) were male and 13 (38.2%) were female while 25 (73.5%) were married and 9 (26.4%) were unmarried. 32 (94.1%) positive HCWs belonged to the undergraduate category while only 2 (5.9%) from the graduate category (Table 2). Of these positive cases, 18 (52.9%) had history of dental treatment, 4 (11.8%) had been transfused, 4(11.6%) had surgery in the past, 4 (11.8%) had a history of hospitalization, 3 (8.82.5) had jaundice and one of them were intravenous drug users (Table 3). HCV RNA was detected in 23 (2.79%) HCWs. The most predominant HCV genotype was 3a (73.91), followed by 3b (13.04%), 1a (4.34%), mixed infection (4.34%) and unidentified genotyping (4.34%). With respect to the professional categories, 44.1% (15 out of 240) of nurses, 32.3% (11 out of 240) of technicians, 14.7% (5 out of 114) of assistant and general staff, 8.8% (3 out of 200) of doctors and none of administrative staff were infected with HCV. In this study occupational accidents most frequently occurred in HCWs by work pressure (38.4%), followed by non-cooperation of patients (30.9%), inappropriate handling of instruments (11.7%), lack of attention (6.9%), hurry (4.4%), inexperience (4%) and others (3.7%).

**Table 2:** HCWs distribution (%) according to gender, marital status, education, age and employment duration.

Variables	Description	No. of total HCWs (%)	No. of positive HCWs (%)
Gender	Male	493 (59.8)	21 (61.76)
		
	Female	331 (40.2)	13 (35.29)

Marital status	Married	677 (82.2)	25 (73.52)
		
	Unmarried	147 (17.8)	9 (26.47)

Education	undergraduate	572 (69.4)	32 (94.11)
		
	Postgraduate	252 (30.6)	2 (5.88)

Age (years)	20-29	195 (23.6)	4 (11.76)
	
	30-39	340 (41.3)	10 (29.41)
	
	40-49	237 (28.8)	17 (50)
	
	50-59	52 (6.3)	3 (8.82)

Employment Duration(years)	1- 5 yr	152 (18.44)	4 (11.8)
	
	6-10 yr	181 (21.96)	5 (14.7)
	
	11-15 yr	216 (26.21)	11(32.4)
	
	16-20 yr	135 (16.38)	7 (20.5)
	
	21-25 yr	91 (11.04)	4 (11.8)
	
	26-29 yr	49 (5.94)	3 (8.8)

**Table 3:** Statistical analysis of HCV positivity in studied population

Variables	HCV Negative		HCV Positive		Chi-square (yates test)
	N	%	N	%	
Male	472	59.7	21	6.17	*P *= 1.000
	
Female	318	40.3	13	38.23	

Married	652	82.5	25	73.53	*P *= 0.2654
	
Unmarried	138	17.5	9	26.47	

H/O blood transfusion	20	2.53	4	11.8	*P *= 0.0089

H/O dental treatment	82	10.4	18	52.9	*P *< 0.0001

H/O surgery	27	3.41	4	11.8	*P *= 0.0409

H/O hospitalization	94	11.9	4	11.8	*P *= 0.9811

H/O jaundice	37	4.68	3	8.82	*P *= 0.489

H/O intravenous drug user	1	0.13	0	0	*P *= 1.000

H/O = history of

A total of 729 occupational exposures were recorded in 572 HCWs during the previous one year period of their service. Of the 572 HCWs who acquired injures, 273 (82.5%) were female and 299 (60.6%) male. The mean age of these HCWs was 30 ± 7.89 years, with a minimum age of 20 years and maximum of 54 years. When the number of occupation exposure among the HCWs of different occupational group participated were compared, a high percentage of nurses sustained injuries (47.3%), followed by technicians (31.3%), doctors (13.1%) and assistant staff (8.2%).

All of HCWs were aware of the importance of screening of HCV. Of the total, only 202 (24.5%) were aware of their viral status while only six knew of their condition as an HCV carrier and twenty eight HCWs with positive results were considered asymptomatic.

## Discusion

The HCV prevalence studies carried out in our country during past decades had limited geographical scope, different time frames, applied diverse methodologies, and predominantly focused on hospital based studies and high risk population groups. Despite considerable diversities and limitations, these studies reported the high prevalence of HCV, promulgating the high burden this viral hepatitis poses to population's health [12]. In these hospitals setting even though, preventive measures were already inexistence, but they were improved after the identification of hepatitis as serious occupational hazard. Due to absence of good preventive measures in the past e.g. extensive reuse of non-sterilized syringes, fragile health structure, unscreened blood transfusion, use of contaminated razor by barber, general poverty and poor education, a high prevalence rate was demonstrated in general population of Pakistan [6,7,9]. The wealth of evidences showed that all these factors appears to have played the predominant role in occupational transmission of HCV [6,9]. Rate of seroprevalence of HCV antibodies in the general population of Pakistan have been reported as 5.31%-7.5% while 4.1 to 36% reported from various parts of Khyber Pakhtunkhwa Pakistan [6].

In this study 4.1% anti-HCV positivity was recorded. This figure was lower to that reported from Rawalpindi (5.6% out of 250) [13], from Karachi (6% out of 217) [14] and from Abbottabad (5.6% out of 125) [10]. The difference in low prevalence rate in this study and high prevalence in previously published literatures from diverse regions of Pakistan reflects the variation in the distribution of HCV between and within different Pakistani geographical areas, actual difference in risk at different hospitals, and the compulsion of mandatory screening of serological status of patients in these hospitals before undergoing invasive procedures (surgery/dental), which alerts the HCWs to facilitate appropriate planning during treating the infected patients. On the other hand attempts of viral screening of blood donors have markedly reduced the transfusion-related infections rate in patient population [5]. In contrast, current figure was higher than mentioned (1.6% out of 383) from Islamabad [8] which could be due to reason that 41% of HCWs had service length between 1-5 years. However, methodological differences of sampling strategies and published data of relatively small-scale surveys also contribute to these differences in seroprevalence.

Genotype-3a was the most prevalent genotype in this studied population followed by genotypes 3b, 1a and mixed infection. It is reported that 75%-90% of HCV Pakistani patients [9] were harboring genotype 3a, also confirmed [5-7,15,16].

As for demographic data, neither age group nor marital status was responsible for any statistically significant differences between both groups for HCV while significant association was found between duration of employment in a clinical environment, age of HCWs and education of HCWs [8,17-19].

Rampal et al., [20] documented that each year an estimated figure of more than three million HCWs experience an injury with a biological contaminated sharp instrument and these exposures result in about 16,000 infections of HCV. In this study prevalence of injuries is not uniform in different working departments, among the professional groups, the nurses had the increased vulnerability of injury as they spent greater amount of time in administrative therapies and everything concerning direct assistance to patients. Mass of same evidence was shown by various workers [1,21-24]

While laboratory technicians, who manipulate microorganisms in laboratory, which might involves incidences like transferring blood from the syringe to the vial and missing the target, and anesthesia technicians posed a great threat of acquisition of hepatitis, also confirmed by different authors from worldwide [20-24]. Frequency among doctors was relatively low, probably due to benefit sufficiently from preventative measures and preoperative screening of patients for HCV in studied hospitals before undergoing surgery. Same conclusion also holds true by others [17,25]. None of the administrative staff was found with positive result for HCV, which might be due to their not in direct contact with the patients, but still that factor not eliminate the risk of infection due to small sample size studied. HCWs were at high risk of encountering occupational injuries to blood and other infectious body fluid and therefore have more chances to acquire HCV in their work places. All the HCV positive HCWs at PIMS hospital, Islamabad, had a definite history of needle stick injury except one had history of surgery [8].

This study results showed that although all HCWs were aware about the importance of screening for hepatitis as it made aware of self-care, start therapy and also apply appropriate preventive measure during their provision of services, however there were gaps in their knowledge and practice. The figures in this study were lower than those reported (93.7%) [8]. In spite of considering important, this approach is flawed for a number factors including ethical, legal, economic, moral, and cultural come into play here. The absence of a prophylactic vaccine from HCV infection, the logic of this approach would dictate that repeat and continual screening for HCV is recommended [19]. Another malaise in our health system is the reuse of contaminated needles and equipment in health related procedure. In this study history of dental treatment, history of surgery and blood transfusion has been demonstrated to be responsible as a route of transmission of HCV. This finding verified results of the earlier studies [6,25,26]. Current study was in agreement with previous study [5,6,9,26] that the major contributing factors towards increased HCV prevalence include unchecked blood transfusions and reuse of injection syringes in Pakistani population, as several small groups involved in recycling and repacking of used un-sterilized syringes, which were easily available in market at low cost. Screening in the long term, reduce the pool of hepatitis-infected HCWs performing exposure prone procedure, consequently reduce the frequency of HCW to patient transmission events [10,21].

In Pakistan, sero-frequency figures are significantly higher (P < 0.0001) for HCV as compared to populations in surrounding countries like India, Nepal, Iran and Afghanistan. On the basis of above unfavorable figure, Pakistan ranked among the top position in prevalence rate in both populations, which is alarming, confirmed by others [5,7]. As persistence of hepatitis infection has grave consequences and no satisfactory treatment is available so far, it will be fast growing gargantuan proportion if special precautions will not take to check its transmission in hospital setting. Therefore the use of preventive precautions is important tools to save themselves of this growing menace. The focus was needed on the safety educational training programs to all level of HCWs and it should be emphasized that there is need to maintain utmost care regarding dealing with needles and sharps and caution during the in-between handling also. Moreover the areas, incidences, trends, activity, procedures and occupational groups that result in a high risk of transmission of hepatitis to HCWs should be identified in a tactful manner, carefully analyzed and will be used to design the preventive strategies for them.

## Conclusion

This study demonstrates that it is necessary to carry out recycling programs addressing issue of universal precautions and proper training of HCWs in the medical field can be pivotal in preventing HCV.

## Abbreviations

HCV: Hepatitis C Virus; HCWs: Health Care Workers.

## Competing interests

The authors declare that they have no competing interests

## Authors' contributions

SK and SA designed and gave a critical view of manuscript writing. SA collected epidemiological data; SNK performed PCR analysis and analyzed the data statistically. SS helped SA in molecular genotyping assays. MB and SS gave critical view of manuscript writing and participated in data analysis. All the authors' read and approved the final manuscript.
